# *Mycobacterium avium* subsp. *paratuberculosis (Map)* Fatty Acids Profile Is Strain-Dependent and Changes Upon Host Macrophages Infection

**DOI:** 10.3389/fcimb.2017.00089

**Published:** 2017-03-21

**Authors:** Marta Alonso-Hearn, Naiara Abendaño, Maria A. Ruvira, Rosa Aznar, Mariana Landin, Ramon A. Juste

**Affiliations:** ^1^Department of Animal Health, NEIKER-Basque Institute for Agricultural Research and Development, Technological Park of BizkaiaDerio, Spain; ^2^Spanish Type Culture Collection (CECT), University of Valencia, Parc Científic Universitat de ValènciaPaterna, Spain; ^3^Department of Pharmacology, Pharmacy and Pharmaceutical Technology, University of SantiagoSantiago de Compostela, Spain; ^4^Servicio Regional de Investigación y Desarrollo Agroalimentario, Agri-Food Research and Development Regional ServiceVillaviciosa, Spain

**Keywords:** *Mycobacterium avium* subp. *paratuberculosis*, *Map*-host interaction, fatty acids, lipid metabolism, macrophages

## Abstract

Johne's disease is a chronic granulomatous enteritis of ruminants caused by the intracellular bacterium *Mycobacterium avium* subsp. *paratuberculosis* (*Map*). We previously demonstrated that *Map* isolates from sheep persisted within host macrophages in lower CFUs than cattle isolates after 7 days of infection. In the current study, we hypothesize that these phenotypic differences between *Map* isolates may be driven be the fatty acids (FAs) present on the phosphadidyl-1-*myo*-inositol mannosides of the *Map* cell wall that mediate recognition by the mannose receptors of host macrophages. FAs modifications may influence *Map*'s envelope fluidity ultimately affecting pathogenicity. To test this hypothesis, we investigated the responses of two *Map* isolates from cattle (K10 isolate) and sheep (2349/06-1) to the bovine and ovine macrophage environment by measuring the FAs content of extracellular and intracellular bacteria. For this purpose, macrophages cell lines of bovine (BOMAC) and ovine (MOCL-4) origin were infected with the two isolates of *Map* for 4 days at 37°C. The relative FAs composition of the two isolates recovered from infected BOMAC and MOCL-4 cells was determined by gas chromatography and compared with that of extracellular bacteria and that of bacteria grown in Middlebrook 7H9 medium. Using this approach, we demonstrated that the FAs composition of extracellular and 7H9-grown bacteria was highly conserved within each *Map* isolate, and statistically different from that of intracellular bacteria. Analysis of FAs composition from extracellular bacteria enabled the distinction of the two *Map* strains based on the presence of the tuberculostearic acid (18:0 10Me) exclusively in the K10 strain of *Map*. In addition, significant differences in the content of Palmitic acid and cis-7 Palmitoleic acid between both isolates harvested from the extracellular environment were observed. Once the infection established itself in BOMAC and MOCL-4 cells, the FAs profiles of both *Map* isolates appeared conserved. Our results suggest that the FAs composition of *Map* might influence its recognition by macrophages and influence the survival of the bacillus within host macrophages.

## Introduction

*Mycobacterium avium* subsp. *paratuberculosis* (*Map*) is the etiological agent of Johne's disease (JD) or paratuberculosis, a chronic granulomatous enteritis of ruminants. *Map* isolates can be classified in two genotypes based on culture characteristics and genome analysis: sheep isolates (also called “type S” or “type I”) and cattle isolates (also called “type C” or “type II) (Collins et al., 1990; Bryant et al., 2016). JD causes considerable economic losses to the livestock and associated industries, due to decreased milk production, premature culling, and reduced carcass value (Ott et al., 1999). This, coupled with an association of *Map* with Crohn's disease and diabetes type I has resulted in an increasing interest in studying JD pathogenesis (Feller et al., 2007; Scanu et al., 2007; Abubakar et al., 2008; Juste et al., 2008, 2009; Naser et al., 2014). Macrophages primarily use the mannose receptor (MR, CD207) as well as the complement 3 receptor for the phagocytosis of *Map* (Souza et al., 2007). These receptors are distinguished by the fact that they mediate the engulfment of microbes without necessarily inciting a proinflammatory immune response and thereby have long been postulated to enhance early intracellular survival of some microbes. Continued efforts to define the factors affecting the early interaction between *Map* and host macrophages are necessary to further our understanding of the pathogenesis of paratuberculosis and final disease outcome. This ongoing research might help in the development of better control strategies and diagnostic techniques.

Not only susceptibility of the host but also virulence properties of Mycobacteria contribute to their pathogenicity. Mycobacteria are characterized by a complex cell wall structure rich in lipids that constitute up to 60% of the dry weight of the organism and that is responsible for many of its unique properties (acid fast staining and resistance to many antibiotics). Among the cell-envelope components, phophatidyl-1-*myo*-inositol mannosides (PIMs) are found in abundant quantities in the cell envelope of all *Mycobacterium* species (Kolattukudy et al., 1997). PIMs are considered not only essential structural components of the cell envelope but also the precursors of the two major mycobacterial lipoglycans, lipomannan (LM) and lipoarabinomannan (Man-LAM). PIMs are based on a phosphadidyl-*myo*-inositol (PI) lipid carrying one to six α-D-Man*p* residues (PIM_1_ to PIM_6_) and up to four acyl chains, with tri- and tetra-acylated PIM dimannoside (Ac_1_PIM_2_,Ac_2_PIM_2_) and PIM hexamannoside (Ac_1_PIM_6_,Ac_2_PIM_6_) as the most predominant classes of PIMs found in *Mycobacterium* species (Guerin et al., 2010; Albesa-Jové et al., 2016). In *Mycobacterium tuberculosis* (*Mtb*), the FAs composition of the tri-acylated forms of the PIM_S_ includes two Palmitic acids and one Tuberculostearic acid (TBSA) (Gilleron et al., 2003). The tetra-acylated forms of the PIMs are present predominantly as two populations bearing either three Palmitic acids/one TBSA or two Palmitic acids/two TBSAs. The Man-LAM *Mtb* is a bacterial ligand for the entry of *Mtb* into macrophages via the MR, and both the mannose caps and the fatty acids are required for efficient binding (Gilleron et al., 2001; Torrelles et al., 2008). Other major effects attributed to the Man-LAM of *Mtb* in phagocytic cells include induction of a rapid IL-10 expression, suppression of TNF-α and IL12 production, inhibition of apoptosis, inhibition of phagosome-lysosome fusion, suppression of oxygen radicals and nitric oxygen generation (Fratti et al., 2003; Majumder et al., 2008). All of these biological activities are abolished with the loss of the fatty acyl appendages, and acylation of a specific site might be important in the context of PIM presentation (Gilleron et al., 2006). The FAs induce a supramolecular organization of Man-LAM in aqueous solution, resulting in the formation of a 30 nm spherical structure, composed of approximately 450 molecules with the mannose caps exposed at the surface (Rivière et al., 2004). This supramolecular structure allows multipoint attachment of Man-LAM, via mannose caps, to the MR pointing toward the importance of the FAs in the conformation and accessibility of the terminal mannosyl structures (Torrelles et al., 2006; Guerin et al., 2010). Using three dimensional models, Torrelles et al. evaluated how the nature of the FAs in the tetra-acylated PIMs affects its spatial conformation (Torrelles et al., 2008). They found that there was a slight effect on the axis when the fourth fatty acid was substituted with Palmitic acid or Oleic acid relative to TBSA. Altogether, these findings reinforce the idea that changes in the FAs composition of Mycobacteria might impact the spatial conformation of the mannose caps for PIMs recognition by the MR.

We previously tested the ability of 10 isolates of *Map* isolated from domestic (cattle, sheep, and goat) and wildlife animal species (fallow deer, deer, and wild boar) to enter, grow and persist in bovine and ovine macrophages (Abendaño et al., 2013, 2014). Our results demonstrated that the 2349/06-1 isolate of *Map* (type S) from sheep persisted within bovine macrophages in lower CFUs and displayed significantly less growth than the two tested bovine isolates (K10 and 6, type C) after 7 days of infection. Although the variations in the estimated log CFUs at day 7 within ovine macrophages between all the tested isolates were not significant, the bovine isolates proliferated more rapidly than the 2349/06-1 isolate which was observed to minimally decrease in log CFUs over 7 days from baseline. Analysis of the mechanisms through which *Map* interferes with macrophage activation and phagosome maturation has shown that engagement of specific membrane receptors with bacterial ligands is the initiating event. Among the cell envelope components of *Map*, the Man-LAM has been identified as one of the main bacterial ligands involved in phagosome-lysosome inhibition, and as a major virulence factor in determining the capacity of *Map* to persist within bovine macrophages (Souza et al., 2013). Since data from studies with *Mtb* suggested that the type of the fatty acyl groups present in the PIMs affect their conformation and the subsequent interaction of the mannose caps with the MR, in the current study FAs profiling of *Map* isolates was performed.

## Materials and methods

### Cell lines

A SV-40 transformed bovine peritoneal macrophage cell line (BOMAC), was obtained from Judith Stabel (USDA, Ames, IA, US) and maintained as previously described (Stabel and Stabel, 1995). An ovine blood macrophage-like cell line (MOCL-4) was obtained from Michel Olivier (INRA, Nouzilly, France) and cultured as previously described (Olivier et al., 2001).

### *Map* isolates, bacterial culture and preparation of bacterial suspensions

Two *Map* isolates from cattle (*Bos taurus*) and sheep (*Ovis aries*) were selected from the collection of isolates of the Mycobacteria laboratory, NEIKER-Tecnalia. The bovine K10 isolate of *Map*, a sequenced strain recovered from a clinical case of paratuberculosis, was obtained from the American Type Culture Collection. The *Map* 2349/06-1 isolate from sheep was kindly provided by A. C. Coelho. Isolate code, country of isolation, and genotype for each *Map* isolate are summarized in Table 1. Entry and intracellular growth of the K10 and 2349/06-1 isolates of *Map* in bovine and ovine macrophages was previously estimated (Abendaño et al., 2013, 2014) and is presented in Table 1. Both *Map* isolates were maintained as glycerol stock at −80°C (Adúriz et al., 1995; Sevilla et al., 2005, 2007). Aliquots of these glycerol stocks were utilized to directly inoculate all subsequent cultures for use in macrophages infections. The isolates of *Map* selected for our study were grown in T25 tissue culture flasks at 37 ± 1°C in 10 ml of Middlebrook 7 H9 broth (Difco Laboratories, Detroit, MI) supplemented with 10% (v/v) oleic acid-albumin-dextrose-catalase (Becton, Dickinson and Company, Franklin Lakes, NJ), 0.05% (v/v) Tween-80 (Sigma-Aldrch, St Louis, MO) and 2 mg l^−1^ of Mycobactin J (Allied Monitor Inc., Fayette, MO) for 20 days at 37°C. Bacterial cells were harvested by centrifugation at 2000 × g for 20 min in a Beckman Coulter Allegra X-12 centrifuge. Bacterial pellets were washed three times with sterile Hank's balanced salt solution (HBSS), resuspended in 2 ml of HBSS, and the resultant suspension was passed 20 times through a 27-gauge needle, and large aggregates were allowed to settle. After 5 min, an aliquot was taken from the top half of the bacterial suspension and diluted in HBSS to a McFarland standard of 1 (3 10^8^ CFUs/ml) with a Densimat (bioMerieux, Marcy l'Étoile, France). Only the top fraction of the suspension containing dispersed bacteria was used for the infection assays.

**Table 1:** **Isolate code, country, host of origin, IS*1311* PCR-REA, and entry and intracellular growth in bovine and ovine macrophages of each *Map* isolate used in the current study**.

**Isolate**	**Country**	**Host**	**PCR-REA type**	**Bovine macrophages^a^**		**Ovine macrophages^b^**	
				**Entry (%)**	**Growth changes^c^ (n-fold)**	**Entry (%)**	**Growth changes^c^ (n-fold)**
K10	US	Cattle	C	70.91	1.84	65.32	1.63
2349/06-1	Portugal	Sheep	S	61.69	1.31	51.93	0.99

a *According to Abendaño et al. (2013)*.

b *According to Abendaño et al. (2014)*.

c *Growth changes (n-fold) were calculated by dividing the number of log _10_ CFU at day 7 by that at day 0*.

### Infection of BOMAC and MOCL-4 cell lines with *Map*

Confluent monolayers of BOMAC and MOCL-4 cell lines grown in a 125-cm^2^ cell culture flask (Corning Costar, New York, US) at 37°C in a 5% CO_2_ were infected with each of the two *Map* isolates at MOI of 1:10 (cell:bacteria). At 4 h post-infection, the medium was collected, centrifuged at 2000 g for 15 min in a Beckman Coulter Allegra X-12 centrifuge and the resultant pellet containing extracellular bacteria was frozen at −80°C. Cell monolayers were washed twice with 20 ml of HBSB and then treated with 200 μg/ml amikacin (Sigma) in HBSB to kill extracellular bacteria. After 2 h at 37°C, the amikacin was removed, the monolayers were washed twice with HBSS and fresh culture medium was added to the monolayers. After 4 days at 37°C, intracellular bacteria were released by lysing the monolayers with sterile water. Cell debris and nuclear fractions were removed by low-speed centrifugation at 400 g for 5 min at 4°C. The bacterial fraction was recovered from the supernatant after additional centrifugation at 2000 g for 15 min.

### Fatty acid methyl esters (FAMEs) extraction

FAMEs were extracted from bacterial pellets by saponification with sodium hydroxide in methanol, methylated with hydrochloric acid in methanol and then extracted with hexane in methyl-tert-butyl-ether (Sasser, 1990). Briefly, 1 ml of 15% (w/v) NaOH in 50% aqueous methanol was added to each bacterial pellet and incubated at 100°C for 30 min in a water bath. The saponified samples were then cooled in a pan of cold tap water for 2 min, acidified and methylated by the addition of 2 ml of 54% 6 N HCl in 46% methanol, and incubated at 80°C for 10 min in a water bath. This step drops the pH of the solution below 1.5 and cause methylation for the increased volatility in a partially polar column of the FAMEs. The methylated FAMEs were then incubated with 1.25 ml of an organic solvent [hexane:methyl-tert-butyl ether (1:1)]. Each sample was mixed for 10 min and the lower phase was removed with a Pasteur pipette. The upper phase was washed with 3 ml of 0.3 M NaOH to remove both free FAMEs and residual reagents. Following the wash step, the organic phase containing the FAMEs was transferred into a GC vial. FAMEs are more volatile than their respective FAs and therefore more suitable for GC analysis.

### GC analysis

The FAMEs were analyzed by GC using the Agilent 6850 gas chromatographic unit equipped with a crossslinked 5% phenylmethyl silicone-fused silica capillary column (25 m × 0.2 mm, Agilent 19091B-102E), a flame ionization detector and an Agilent 6850 automatic liquid sampler. The column temperature ramped from 170 to 270°C at 5°C min^−1^, then increased to 310°C at 40°C min^−1^ and held for 1.5 min. Hydrogen served as the carrier gas at 0.5 ml min^−1^. An external calibration standard, a mixture of straight chain saturated FAs from 9 to 20 carbons in length (9:0 to 20:0) and five hydroxyl acids (Sherlock MIS Calibration Standard, Part#1200-A, MIDI Inc., Newark, DE, US), was used. The hydroxyl compounds are especially sensitive to changes in pressure/temperature relationships and to contamination of the injection port lines. As a result, these compounds function as real-time quality control checks for the system. Retention time data obtained by injecting the calibration standard is converted to Equivalent Chain Length (ECL) data for bacterial FAs naming. The Retention factor (RFact) for each FA can be derived as a function of its elution time in relation to the elution time of the known series of straight chain FAs. GC allows windows to be set at 0.010 ECL units, giving great resolution of FAs isomers.

FAs analysis using the Sherlock® Microbial Identification software was used to automatically name and quantitate the peaks in an unknown sample (MIDI Inc, Newark, DE, US). The FAMEs extraction procedure may carry over sterols and other non-fatty acid material. Additionally, electronic noise may result in transient spikes, which might interfere with the chromatography. However, FAs peaks always have area/height ratios greater than 0.017 and less than 0.070, making it possible to set exclusionary parameters at these levels. Electronic noise spikes are typically <0.017 and non-fatty acid peaks are usually >0.070, allowing rejection of these artifacts. The Sherlock approach is set to use a “Summed in Feature” wherever imperfect peak separation occurs. Reproducibility of the profiles was calculated by comparing two different FAMEs extracts.

### FAs profile pattern recognition and cluster analysis

The Sherlock® Microbial Identification System with covariance matrix, principal component analysis and pattern recognition software was used to generate FAs profiles for our samples. The covariance matrix takes into account the mole-for-mole relationship of the conversion of one FA to another which might occur in relation to a temperature shift. The pattern recognition uses ratios between FAs amounts in addition to the principal component base. The dendrogram and principal component analysis (2-D plots) programs of the Sherlock® Microbial Identification System use data from FAs analyses to graphically illustrate relationships between the samples. The dendrogram analysis produces unweight pair matching based on FAs composition. In a dendrogram, the Euclidean distance is the distance in n-dimensional space between two bacterial samples when their FAs composition is compared. Lower linkages indicate greater similarity. In addition to dendrogram, the 2-D Plot is another cluster analysis tool which uses a principal components analysis of FAs profiles to group entries in a two dimensional space. The x-axis represents principal component 1, and the y-axis represents principal component 2.

### Statistical analysis

The percentage of 21 FAs in the K10 (type-C) and 2349/06-1 (type-S) isolates of *Map* under different environmental conditions (extracellular, intracellular, or 7H9 medium) were compared with the General Lineal Model (GLM) procedure of the SAS statistical package version 9.3 (SAS Institute Inc., Cary, NC). Differences were considered significant when *P*-values were <0.05. Correlations between FAMEs profiles were examined with the principal components procedure of the SAS software.

### Neurofuzzy logic (NFL) analysis

The percentages of each of the 21 FAs in the K10 (type-C) and 2349/06-1 (type-S) isolates of *Map* under different environmental conditions (BOMAC, MOCL-4), and localizations within the host cell (extracellular and intracellular) were modeled using the NFL FormRules® software v4.03 (Intelligensys Ltd., 2013, Stokesley, UK). The FormRules software contains various statistical fitness criteria including Cross Validation, Minimum Description Length, Structural Risk Minimization, Leave One Out Cross Validation and Bayesian Information Criterion. All were analyzed in order to generate the model with the best predictability together with the simplest and more intelligible rules. The predictability of the model was assessed using correlation coefficient (R^2^) and ANOVA F-values for the percentage of each fatty acid.

$$R^{2}=\left(\frac{1-\sum_{i\;=\;1}^{n}{\left(y_{i}-{y\prime }_{i}\right)}^{2}}{\sum_{i\;=\;1}^{n}{\left(y_{i}-{y\prime \prime }_{i}\right)}^{2}}\right)\times 100\%$$

Where *y*” is the mean of the dependent variable, and the *y*' is the predicted value calculated by the model. ANOVA *F*-values over its critical values for the corresponding degrees of freedom are an indication of reasonable model predictabilities (Colbourn and Rowe, 2009). For each of the generated rule a “membership degree” or confidence level is defined which specifies how a “value” belongs to that fuzzy subset (from 0 to 1).

## Results

### FAs profiles of *Map* isolates grown in middlebrook 7H9 broth

**Figure 1:**
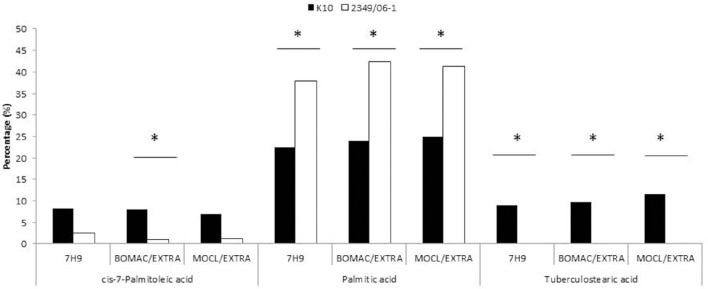
**FAs of the K10 (Type C) and 2349/06-1 (Type S) isolates of *Map* that showed statistically significant differences in abundance between the two isolates**. BOMAC and MOCL-4 cells were infected with the K10 and 2349/06-1 isolates of *Map* at MOI of 1:10. At 4 h p.i., the medium containing the extracellular bacteria was collected, centrifuged at 2000 g for 15 min and the FAMEs of the bacterial pellets extracted and analyzed by GC. FAs of the K10 and 2349/06-1 isolates of *Map* grown in Middlebrook 7H9 medium that showed statistically significant differences in abundance are also included in the figure. Relative amount of each FA for each experimental condition (7H9 medium or extracellular) is presented as the percentage of the total FAs content. Statistically significant differences are indicated with an asterisk.

A representative chromatogram for each *Map* isolate (K10 and 2349/06-1) grown in Middlebrook 7H9 medium is presented in Figure 1S. The identities of 97.51 and 87.87% FAs of the K10 and 2349/06-1 isolates were confirmed respectively by demonstrating retention times similar to those of known standards. The peak, R Factor, systematic name, usual name and relative frequency of each identified FA are summarized in Table 2. FAs found ranged from 9 to 20 carbon atoms. They consisted of nine saturated FAs (9:0, 10:0, 12.0, 14:0, 15:0, 16:0, 17.0, 18:0, 20:0), six monounsaturated FAs (16:1 w9c, 16:1 w7c, 16:1 w6c, 17:1 w8c, 18:1 w9c, 18:1 w7c), one double-unsaturated FA (18:2 w6,9c), one 10-methyl branched of 18 carbon atoms (18:0 10Me) and three Sum in Feature FAs. The Palmitic acid (16:0) and Oleic acid (18:1 w9c) were the most abundant FAs and together represented more than 50% of the total cellular FA content of both *Map* isolates. From the 21 identified FAs, 14 were present in the profiles of both *Map* strains. The K10 isolate was characterized by the presence of four FAs (9:0, cis-10-Palmitoleic acid, Summed in Feature 1, and TBSA) which were absent in the 2349/06-1 isolate. The 16:0 2,4 DiMe was detected in very small amount only in the S-type isolate of *Map*. Statistical analysis of the FAs composition of both *Map* isolates grown in Middlebrook 7H9 medium (Figure 1) enabled the distinction between both *Map* strains based on the presence of the TBSA (18:0 10Me) exclusively in the C-type isolate of *Map*. Among the 14 FAs present in the profiles of both *Map* isolates, significant differences were observed in the abundances of Palmitic acid and TBSA between both *Map* isolates.

**Table 2:** **Peak, R Factor, and FAs analysis of the K10 and 2349/06-1 isolates of *Map* grown in 7H9 medium**.

**Peak**	**Systematic name**	**Usual name**	**RFactor**	**K10**	**2349/06-1**
				**FAs (%)^a^**	
9:0			1.296	0.25	
10:0	Decanoic acid	Capric acid	1.208		
12:0	Dodecanoic acid	Lauric acid	1.054	0.20	0.23
14:0	Tetradecanoic acid	Myristic acid	0.973	3.26	2.75
15:0	Pentadecanoic acid	Pentadecylic acid	0.950	0.54	0.53
16:1 w9c	(7Z)-7-Hexadecenoic acid	cis-7-Palmitoleic acid	0.937	8.16	2.53
16:1 w7c	(9Z)-9-Hexadecenoic acid	Palmitoleic acid	0.937	1.27	3.87
16:1 w6c	(10Z)-10-Hexadecenoic acid	cis-10-Palmitoleic acid	0.936	1.31	
16:0	Hexadecanoic acid	Palmitic acid	0.935	22.54	37.95
17:1 w8c	(9Z)-9-Heptadecenoic acid	cis-Margoreleic acid	0.933		
Sum in Feature 1	16:0 8ME/16:0 10ME		0.930	0.35	
16:0 2,4 DiMe			0.929		0.40
Sum in Feature 2	17:1 w7c/18 Alcohol		0.927	2.23	2.03
17:0	Heptadecanoic acid	Margaric acid	0.925	0.84	0.51
18:2 w6,9c	1,1,-Dimerthoxyoctadecadiene		0.921	0.68	0.63
18:1 w9c	(9Z)-9-Octadecenoic acid	Oleic acid	0.921	28.51	34.23
18:1 w7c	(11Z)-11-Octadecenoic acid	cis-Vaccenic acid	0.920	1.36	1.81
18:0	Octadecanoic acid	Stearic acid	0.919	7.79	6.34
18:0 10Me	10-Methyloctadecanoic acid	TBSA	0.918	8.97	
Sum in Feature 3	20:0 ALC/18.838ECL/19:0 Cycloprop w10c/19:0 Cycloprop w8c		0.916	9.95	4.74
20:0	Icosanoic acid	Arachidic acid	0.909	1.81	1.46

a *Relative amount of each FA is expressed as a percentage of the total FAs content*.

### FAs content of *Map* isolates in the extra- and intracellular environment of BOMAC and MOCL-4 cells

**Figure 2:**
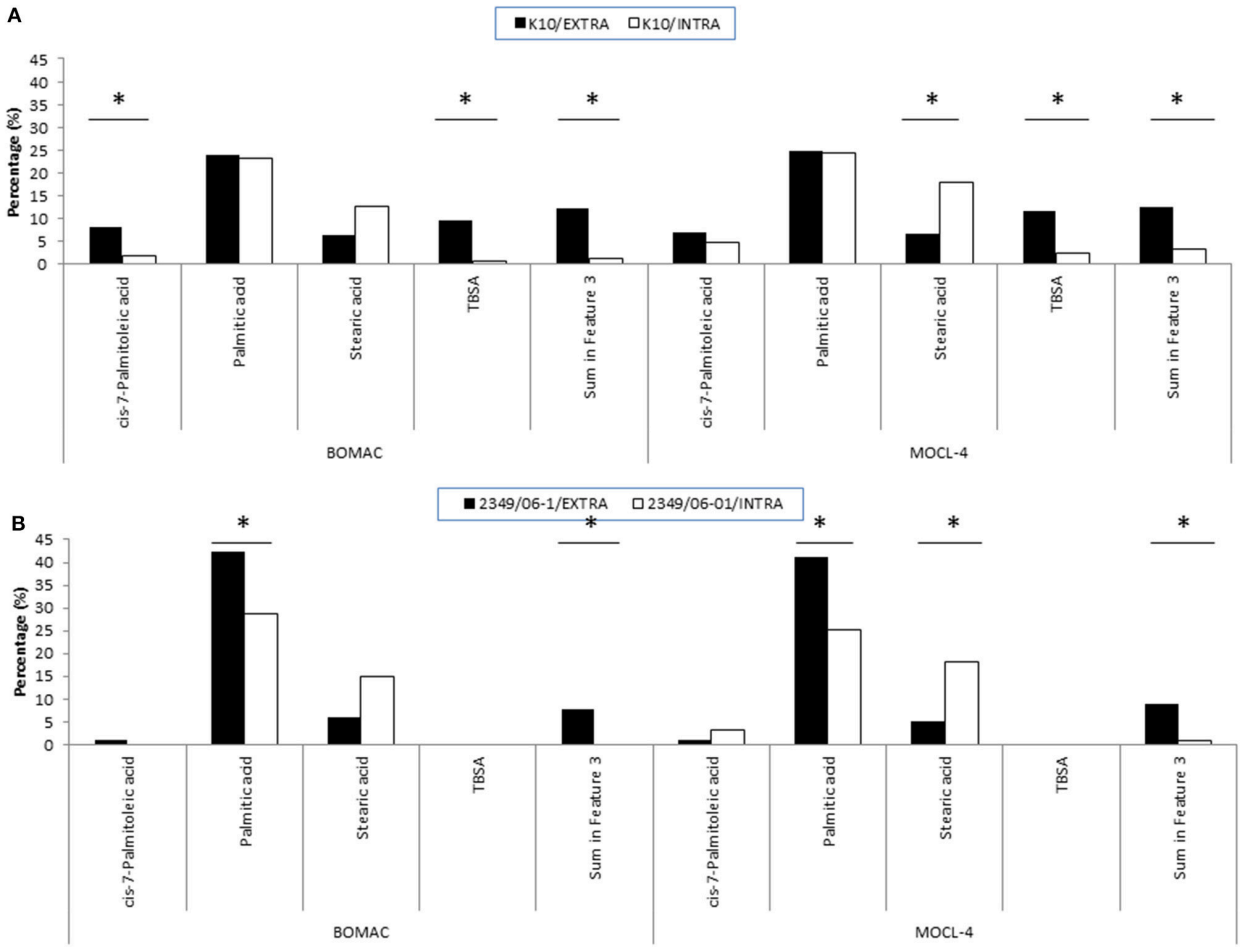
**FAs of the K10 (A)** and 2349/06-1 **(B)** isolates of *Map* that showed statistically significant differences in abundance when the bacteria was recovered from the extra- or intracellular environment of infected BOMAC and MOCL-4 cells. BOMAC and MOCL-4 cells were infected with the K10 and 2349/06-1 isolates of *Map* at MOI of 1:10. At 4 h p.i., the medium containing the extracellular bacteria was collected, centrifuged at 2,000 g for 15 min and the resultant pellet containing extracellular bacteria was frozen at −80°C. The cell monolayers were washed twice with 20 ml of HBSB and then treated with 200 μg/ml amikacin (Sigma) in HBSB to kill extracellular bacteria. After 2 h at 37°C, the amikacin was removed, the monolayers were washed twice with HBSS and fresh culture medium was added to the monolayers. After 4 days at 37°C, the intracellular bacteria were released by lysing the monolayers with sterile water. Cell debris and nuclear fractions were removed by low-speed centrifugation at 400 g for 5 min at 4°C. The bacterial fraction was recovered from the supernatant after additional centrifugation at 2,000 g for 15 min. FAMEs were extracted from the extra and intracellular bacteria and analyzed by GC. Relative amount of each FA for each experimental condition (extracellular or intracellular) is presented as the percentage of the total FAs content. Statistically significant differences are indicated with an asterisk.

**Figure 3:**
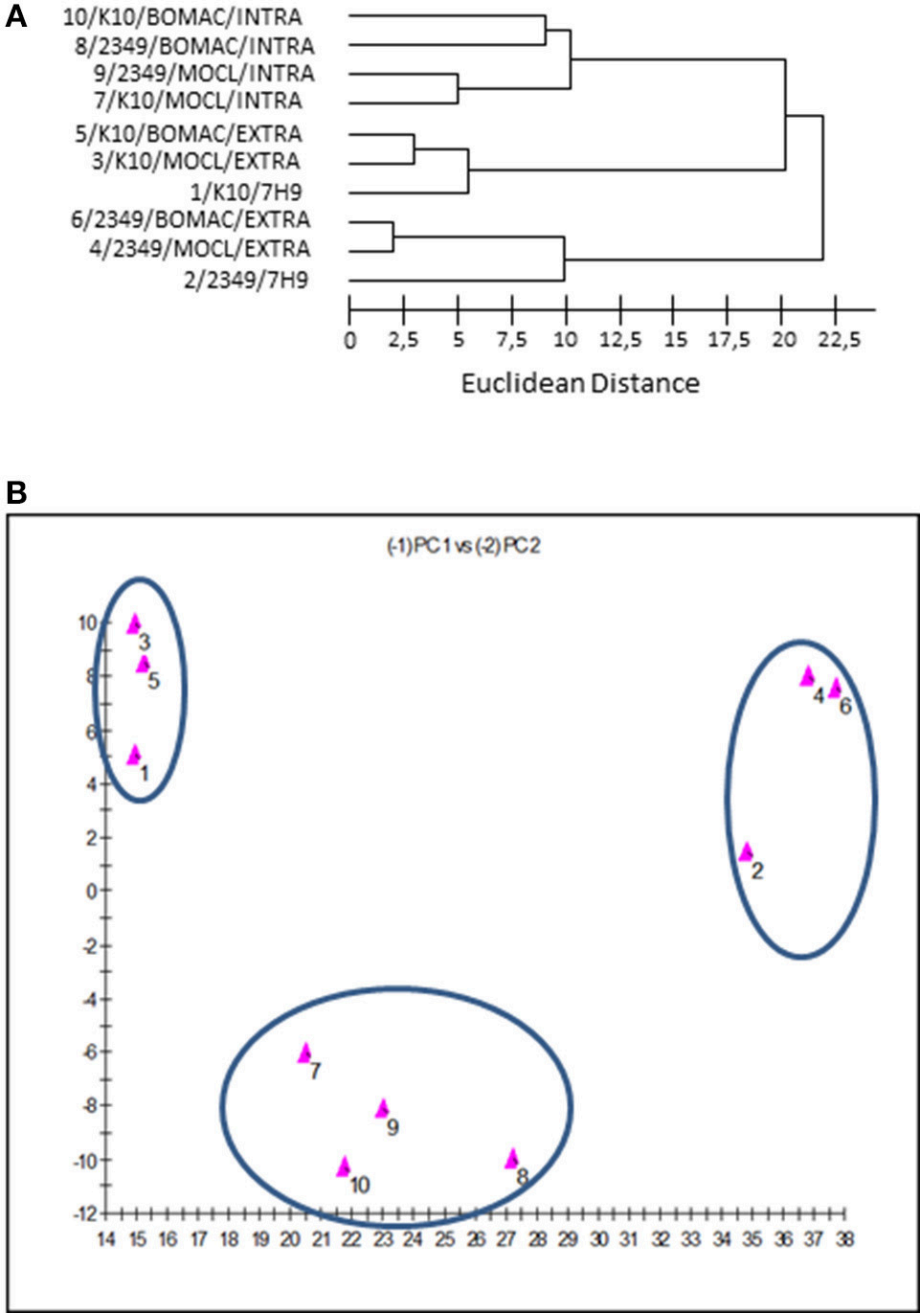
**(A)** Dendrogram generated using the Sherlock Microbial Identification System with the FAs profiles of the K10 and 2349/06-1 isolates of *Map* under the three assessed environmental conditions (extracellular, intracellular, and 7H9 Middlebrook). The Euclidean distance is the distance in n-dimensional space between the bacterial samples when their FAs composition is compared. Lower linkages indicate greater similarity **(B)**. Two dimensional-plot based on principal-component analysis of the FAs profiles of the K10 and 2349/06-1 isolates of *Map* under the three assessed environmental conditions (extracellular, intracellular and 7H9 grown). The x-axis represents principal component 1 (PC1) and the y-axis represents principal component 2 (PC2).

To evaluate whether the extra- and/or the intracellular environment influences *Map* FAs composition, BOMAC and MOCL-4 cells were infected with the K10 and the 2349/06-1 isolates of *Map*. At 4 h post-infection, the medium was collected, centrifuged at 2000 g for 15 min and the FAMEs of the bacterial pellet extracted. After 4 days at 37°C, intracellular bacteria were recovered by differential centrifugation after lysing the infected monolayers. FAMEs were extracted from extra- and intracellular bacteria and analyzed by GC (Figures 2S, 3S). The percentages of the FAs found in both isolates recovered from the extra- and intracellular environment of BOMAC and MOCL-4 cells are reported in Table 3. The FAs profiles of both *Map* isolates grown in 7H9 medium and recovered from the extracellular medium of BOMAC or MOCL-4 cells were found to be highly similar. As shown in Figure 1, the amount of Palmitic acid and TBSA was quite different in both isolates of *Map* recovered from the extracellular environment regardless of the cell line. Significant differences in the content of cis-7-Palmitoleic acid between both isolates of *Map* were only observed in the bacteria recovered from the extracellular environment of BOMAC cells. When comparing the FAs profiles of each *Map* isolate recovered from the extra- or intracellular environment of BOMAC and MOCL-4 we observed that *Map* FAs profiles change upon host macrophages infection and that these changes are strain-dependent (Figure 2). Since the K10 and 2349/06-1 isolates recovered from the extracellular environment showed a different FAs profile they had to re-align their FAs metabolism inside host macrophages in a different manner. Once within host macrophages, FAs profiles of both *Map* isolates were equivalents regardless of the cell line.

**Table 3:** **Comparative FAs profiles of intracellular K10 and 2349/06-1 isolates of *Map* recovered from BOMAC and MOCL-4 cells at 4 d p.i. vs. extracellular bacteria**.

		**BOMAC**				**MOCL-4**			
**Peak**	**Usual name**	**K10 Extra**	**2349 Extra**	**K10 Intra**	**2349 Intra**	**K10 Extra**	**2349 Extra**	**K10 Intra**	**2349 Intra**
		**FAMEs (%)^a^**				**FAMEs (%)^a^**			
9:0			0.35	2.52					1.76
10:0	Capric acid			1.01					0.40
12:0	Lauric acid	0.26		2.38	3.97	0.21		1.33	1.60
14:0	Myristic acid	3.48	2.99	9.49	4.54	3.43	2.75	2.91	2.46
15:0	Pentadecylic acid	0.59	0.67			0.70	0.73		0.64
16:1 w9c	cis-7-Palmitoleic acid	8.02	1.07	1.73		7.01	1.13	4.67	3.38
16:1 w7c	Palmitoleic acid	1.17		2.10	4.10	0.97		3.08	2.87
16:1 w6c	cis-10-Palmitoleic acid	1.74	4.09			1.48	4.27		
16:0	Palmitic acid	24.02	42.35	23.30	28.64	24.83	41.28	24.38	25.37
17:1 w8c	cis-Margoreleic acid			0.93					2.35
Sum in Feature 1		0.51				0.66			
16:0 2,4 DiMe			0.47				0.45		
Sum in Feature 2		2.49	2.86			2.52	2.98		
17:0	Margaric acid	0.90	0.49			1.03	0.50	2.66	2.94
18:2 w6,9c		0.54		4.31	5.53	0.44		3.42	4.17
18:1 w9c	Oleic acid	25.69	28.10	30.93	32.28	23.84	28.84	30.25	29.77
18:1 w7c	cis-Vaccenic acid	1.05	1.74	5.71	6.03	0.84	1.55	2.40	2.47
18:0	Stearic acid	6.28	5.96	12.64	14.91	6.52	5.30	17.90	18.20
18:0 10Me	TBSA	9.66		0.55		11.51		2.44	
Sum in Feature 3		12.24	7.82	1.29		12.52	9.08	3.27	0.84
20:0	Arachidic acid	1.37	1.04	1.10		1.47	1.13	1.29	0.78

a *Relative amount of each FA is expressed as a percentage of the total FAs content*.

### Clustering analysis

The dendrogram and 2D-plots derived from the FAs profiles of both isolates under the three assessed environmental conditions (extra, intra, and 7H9 grown) are presented in Figures 3A,B, respectively. FAs profiles were clustered into three groups. The first group consisted of the FAs profiles of the K10 and 2341/06-1 isolates recovered from the intracellular environment of BOMAC and MOCL-4 cells. The FAs profiles in the first group were clustered into two subgroups according to subtle differences of FAs depending of the host cell line.

The second group includes the FAs profiles of the K10 strain grown in 7H9 medium or recovered from the extracellular medium of infected BOMAC or MOCL-4 cells. The common feature of this group is that the amount of Palmitic acid and TBSA was different than the 2341/06-1 isolate which was clustered in a third group. Although the extracellular and 7H9-grown bacteria belonged to the same cluster, they formed two separate subgroups that reflected environmental divergence. The FAs profiles of each isolate of *Map* recovered from the extracellular environment of BOMAC and MOCL-4 cells were very similar, and the analysis could not differentiate these two extracellular environments. An excellent correlation between the two-dimensional plots obtained with the principal components procedures of the Sherlock Microbial Identification System and the SAS software was obtained.

### Neurofuzzy logic (NFL) analysis

**Figure 4:**
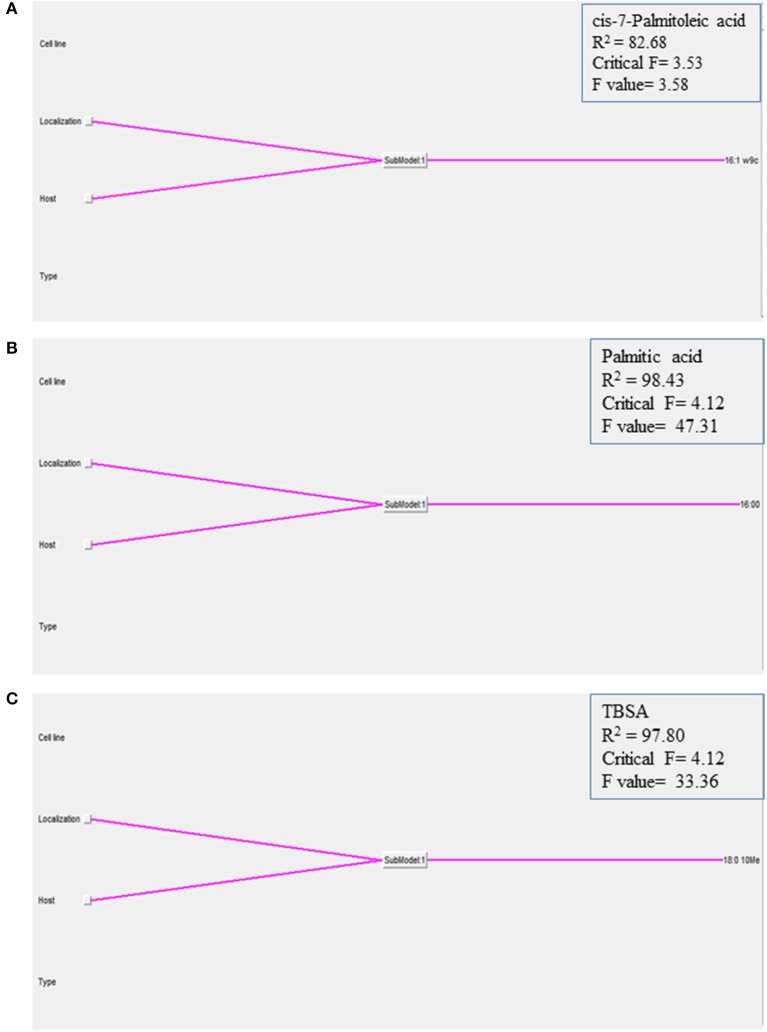
**Graphical representation of the NFL models generated for the (A)** cis-7-Palmitoleic acid, **(B)** Palmitic acid, and **(C)** TBSA; the percentages of each of the 21 identified FAs in the K10 and 2349/06-1 isolates of *Map* recovered from two localizations (extra- and intracellular) of two host cell lines (BOMAC and MOCL-4) were modeled using the NFL FormRules software v4.03. The effect of the specific *Map* isolate (bovine or ovine) and its localization within the host cell (extracellular or intracellular) determined the amount of the three presented *Map* FAs. The predictability of each submodel was assessed using correlation coefficient (R^2^) and ANOVA *F*-values for the percentage of each FA. ANOVA *F*-values over its critical values are an indication of reasonable model predictabilities.

In order to understand how the different environmental conditions (BOMAC, MOCL-4), and localization within the host cell (extra- and intracellular) contribute alone or in combination to the amount each of the 21 FAs identified in the K10 and 2349/06-1 isolates of *Map*, our data were modeled using the NFL FormRules® software v4.03 (Intelligensys Ltd., 2013, Stockesley, UK). Within the statistical fitness criteria included in this software, Structural Risk Minimization was selected to give our model with the best predictability and, simultaneously, the simplest and more intelligent rule sets. This approach allowed the discrimination of the inputs (host of origin of each isolate, genotype, cell line, and localization) which more accurately explained the variability of the amount of each identified FA. For instance, the effect of the *Map* isolate (K10 or 2349/06-1) and its localization within the host cell (extra- or intracellular) determined the variability in the amount of cis-7-Palmitoleic acid, Palmitic acid and TBSA (Figure 4). NFL technology generated a set of “IF…THEN” rules per submodel with their corresponding membership degrees (Table 4). These rule sets represent the cause-effect relationships of the different inputs on the percentage of each FA. For example, for Rule 9: “If the isolate is the K10 and if it is localized in the extracellular environment of the host cell then the amount of TBSA is high with a confidence level of membership of 0.92.” In contrast, if the isolate is the 2349/06-1 the amount of TBSA is low with a confidence level of membership of 1.00 (Rule 10). Interestingly, the only factor explaining the variability in the amount of the Stearic acid and Sum in Feature 3 of *Map* was the bacterial localization, extra- or intracellular.

**Table 4:** **Rules generated by the neurofuzzy logic model**.

**Fatty acid**	**Rule**	***Map*** **isolate**	**Localization**	**Percentage (%)**	**Confidence level**
**16:1 w9c**					
cis-7 Palmitoleic acid	1	K10	Extra	HIGH	0.94
	2	2349/06-1	Extra	LOW	0.86
	3	K10	Intra	LOW	0.60
	4	2349/06-1	Intra	LOW	0.79
**16:0**					
Palmitic acid	5	K10	Extra	LOW	0.94
	6	2349/06-1	Extra	HIGH	0.97
	7	K10	Intra	LOW	0.97
	8	2349/06-1	Intra	LOW	0.81
**18:0 10Me**					
Tuberculostearic acid	9	K10	Extra	HIGH	0.92
	10	2349/06-1	Extra	LOW	1.00
	11	K10	Intra	LOW	0.87
	12	2349/06-1	Intra	LOW	1.00
**18:0**					
Stearic acid	13		Extra	LOW	0.94
	14		Intra	HIGH	0.82
**SUM IN FEATURE 3**					
	15		Extra	HIGH	1.00
	16		Intra	LOW	1.00

## Discussion

Much of what is known of the roles of PIMs in Mycobacteria-host interaction is derived from *in vitro* studies using various cell models and purified PIMs molecules or whole mycobacterial cells (Guerin et al., 2010; Torrelles and Schlesinger, 2010). In *Mtb*, the nature of its FAs has been shown to impact the spatial conformation of the PIMs mannose caps for recognition by the MR, the host immune response and the fate of the bacillus within human macrophages (Torrelles et al., 2008). In the current study, comparative FAs profiling was performed to detect differences between a bovine and an ovine isolate of *Map* before and after entry within macrophages. FAs analysis allows us to consider not only the presence or absence of each FA but also use the data in a quantitative fashion. Previously, a set of 10 FAs (14:0, 16:1w7c, 16:1w6c, 16:0, 18:2w6,9c, 18:1w9c, 18:0, 18:0 10 Me, Summed in Feature 2 and Summed in Feature 3) were found in five *Mycobacterium avium* complex strains, which were differentiated from *Mtb* and *Mycobacterium xenopi* strains by the presence of Palmitoleic acid (16:1 w7c), and Summed in Feature 2 and 3 (Ozbek and Aktas, 2003). As a result, it was suggested that these three FAs could be used as markers to identify and distinguish *Mycobacterium avium* complex strains from other Mycobacteria. Our results agreed with these observations because the Palmitoleic acid, Summed in Feature 2 and 3 were consistently identified in the two *Map* isolates tested in the current study.

The percentage of the 21 identified FAs in the K10 and 2349/06-1 isolates of *Map* under different environmental conditions (extracellular, intracellular, or 7H9 medium) were used to generate a statistical GLM. The GLM provided evidence that the lack of TBSA and a significant increased Palmitic acid content correlated with the significant decrease of survival of the 2349/06-1 isolate of *Map* within macrophages when compared with the K10 strain. Our data was also modeled using the NFL technology which is able to model complex non-linear relationships hidden in data, having a higher accuracy in prediction than classical statistics and helping the understanding of the complex relationships between variables (Shao et al., 2006; Landín et al., 2009; Gago et al., 2010). The rule sets generated with the logic model were in agreement with the findings based on statistical analysis confirming differences in the FAs profiles of both isolates of *Map* in the extracellular environment of the host cell. Previously, a slight effect on the axis of the Ac_2_PIM_6_ of *Mtb* was observed when the fourth FA was substituted with Palmitic acid or Oleic acid relative to TBSA (Torrelles et al., 2008). Accordingly, we suggest that the lack of TBSA or its replacement may affect the ability of *Map* to infect and to survive within host macrophages. Subtle changes in the FAs composition of *Map* might alter: (i) the amount of Man on the cell surface, and/or (ii) might cause a different spatial conformation, disposition and/or localization of Man within the cell envelope of *Map*.

TBSA is a lipid tail of PMIs reported as a constituent of the cell wall of the genus *Mycobacterium*, including *Mtb* (Odham et al., 1979; Lambert et al., 1986) and other phylogenetically related organisms within the suborden Corynebacterineae including the genera *Nocardia, Corynebacterium, Gordonia*, and *Turicella* but not in mammalian hosts (Luquin et al., 1991). For this reason, the detection of TBSA with GC has been used for rapidly diagnosing pulmonary *Mtb* infection (Cai et al., 2013; Dang et al., 2015). Apart from *Mycobacterium gordonae*, the other species of low pathogenic *Mycobacterium* that has been reported to lack TBSA is *Mycobacterium leprae* (Asselineau et al., 1981; Chiodini and Van Kruiningen, 1985). In our study, we found that the TBSA was present in the K10 strain of *Map* (type C) but consistently absent in the 2349/06-1 isolate (type S). Therefore, we propose that this FA could be used as a suitable chemical marker for discrimination between the two genotypes of *Map*. However, we recognize that further studies with multiple isolates of the same host origin and of different genotypes are needed to sustain this assumption.

The ability to remodel the bacterial cell wall in accordance with the changing environmental conditions (i.e., temperature, osmolarity, pH, and concentrations of specific ions) is an essential adaptative strategy for bacterial pathogens. Gram-negative bacteria with both environmental and mammalian reservoirs can synthesize modified forms of lipid A, the biologically active component of the lipopolysaccharide of the outer membrane, in response to environmental stimuli and temperature change (Li et al., 2012). Even small modifications to the outer membrane composition, such as shortening/lengthening acyl chains components of the Lipid A, can alter the bacterium's outer membrane integrity, immune stimulation and pathogenesis. Previous studies highlighted a response of *Mtb* to the intramacrophage conditions by upregulating genes involved in lipid degradation or inhibiting lipid biosynthesis (Betts et al., 2002; Rengarajan et al., 2005; Mukhopadhyay et al., 2012). As *Mtb, Map* adapts to the intracellular environment in the macrophage, where the bacteria overcome exposure to cationic antimicrobial peptides, reactive oxygen species (ROS) and nutrient starvation, via the regulation of genes affecting its envelope's composition (Thirunavukkarasu et al., 2014). In fact, a different lipid profile was previously observed in the envelope of intracellular *Map* after 1 h of infection (Alonso-Hearn et al., 2010; Everman et al., 2015). The full significance of these alterations in lipid metabolism had to be yet revealed to identify the issues facing *Map* within host macrophages. In addition, little data is available about how *Map* modulates FAs metabolism in response to the macrophage environment and whether this modulation is strain-specific and/or environmental-specific. Our study revealed that *Map* FAs composition changes upon macrophages infection and that these changes are strain dependent. Once within host macrophages, the intracellular FAs profiles of both *Map* isolates were equivalents regardless of the macrophage origin, bovine or ovine. Our findings support the idea that maintaining a particular FAs composition might confer certain advantage within the host cell environment.

In conclusion, our results provide evidence that the clinical spectrum of paratuberculosis may be dictated by the surface exposed FAs defined by each *Map* strain. The K10 strain that had more TBSA and less Palmitic acid than the 2349/06-1 isolate was highly successful in establishing an infection in macrophages likely by having a more favorable PIMs engagement of the MR on macrophages. Since the TBSA is a lipid tail of PIMs exclusively found in the cell wall of the genus *Mycobacterium*, we can conclude that structural modifications of the PI anchor may represent a mechanism for mycobacteria to gain advantage in establishing infection. Moreover, we show that the FAs profiles of two different *Map* isolates change during host cell infection and provide novel insights into the metabolic adaptation of *Map* within host macrophages. Our study provides new targets for derivation of attenuated *Map* strains by targeted gene disruption and reveals the intrincate connection between metabolism and virulence in *Map*.

## Author contributions

MAH designed the study and drafted the manuscript. NA conducted Map infection experiments. MR and RA performed FAMEs extraction, GC, and FAMEs pattern recognition and cluster analysis. RJ and ML analyzed the FAs database and generated the GLM and the NFL models, respectively. All the authors helped in the interpretation of the obtained data, revised the manuscript critically for important intellectual content and gave the final approval of the version to be published. In addition, all the authors agreed to be accountable for all aspects of the work in ensuring that questions related to the accuracy or integrity of any part of the work are appropriately investigated and resolved.

## Funding

Financial support for this work was provided by grants from the Instituto Nacional de Investigación y Tecnología Agraria y Alimentaria (INIA) and by European Funds for Regional Development (FEDER) (RTA2011-00049; RTA2014-00009) to MAH. Additional support is provided by a grant from the Xunta of Galicia (ED431C 2016/008). NA had a fellowship from the department of Agriculture of the Basque Government.

### Conflict of interest statement

The authors declare that the research was conducted in the absence of any commercial or financial relationships that could be construed as a potential conflict of interest.

## Abbreviations

*Map*: *Mycobacterium avium subsp. paratuberculosis*
FAs: fatty acids
MR: mannose receptor
PIMs: phosphadidyl-1-*myo*-inositol mannosides
LM: lipomannan
Man-LAM: lipoarabinomannan
PI: phosphadidyl-*myo*-inositol
TBSA: tuberculostearic acid
*Mtb*: *Mycobacterium tuberculosis*
GC: gas chromatography
HBSS: Hank's balanced salt solution
FAMEs: Fatty Acid Methyl Esters
ECL: Equivalent Chain Length
GLM: General Lineal Model
NFL: Neurofuzzy logic.

## References

[B1] Abendaño N. Sevilla I. A. Prieto J. M. Garrido J. M. Juste R. A. Alonso-Hearn M. (2013). *Mycobacterium avium* subspecies *paratuberculosis* isolates from sheep and goats show reduced persistence in bovine macrophages than cattle, bison, deer and wild boar strains regardless of genotype. Vet. Microbiol. 163, 325–334. 10.1016/j.vetmic.2012.12.042 23415474

[B2] Abendaño N. Tyukalova L. Barandika J. F. Balseiro A. Sevilla I. A. Garrido J. M.. (2014). *Mycobacterium avium* subsp. *paratuberculosis* isolates induce *in vitro* granuloma formation and show succesful survival phenotype, common anti-inflammatory and antiapoptotic responses within ovien macrophages regardless og genotype or host of origin. PLoS ONE 9:e104238. 10.1371/journal.pone.0104238 25111300 PMC4128652

[B3] Abubakar I. Myhill D. Aliyu S. H. Hunter P. R. (2008). Detection of *Mycobacterium avium* subspecies *paratuberculosis* from patients with Crohn's disease using nucleic acid-based techniques: a systematic review and meta-analysis. Inflamm. Bowel Dis. 14, 401–410. 10.1002/ibd.20276 17886288

[B4] Adúriz J. J. Juste R. A. Cortabarria N. (1995). Lack of mycobactin dependence of mycobacteria isolated on Middlebrook 7H11 from clinical cases of ovine paratuberculosis. Vet. Microbiol. 45, 211–217. 7571372 10.1016/0378-1135(95)00037-b

[B5] Albesa-Jové D. Svetlíková Z. Tersa M. Sancho-Vaello E. Carreras-González A. Bonnet P.. (2016). Structural basis for selective recognition of acyl chains by the membrane-associated acyltransferase PatA. Nat. Commun. 7:10906. 10.1038/ncomms10906 26965057 PMC4792965

[B6] Alonso-Hearn M. Eckstein T. M. Sommer S. Bermudez L. E. (2010). A *Mycobacterium avium* subsp. *paratuberculosis* LuxR regulates cell envelope and virulence. Innate Immun. 16, 235–247. 10.1177/1753425909339811 19710090

[B7] Asselineau C. Clavel S. Clément F. Daffé M. David H. Lanéelle M. A.. (1981). Lipidic constituents of “*Mycobacterium leprae*” isolated from experimentally infected armadillo. Ann. Microbiol. 132A, 19–30. 7020522

[B8] Betts J. C. Lukey P. T. Robb L. C. McAdam R. A. Duncan K. (2002). Evaluation of a nutrient starvation model of *Mycobacterium tuberculosis* persistence by gene and protein expression profiling. Mol. Microbiol. 43, 717–731. 10.1046/j.1365-2958.2002.02779.x 11929527

[B9] Bryant J. M. Thibault V. C. Smith D. G. McLuckie J. Heron I. Sevilla I. A.. (2016). Phylogenomic exploration of the relationships between strains of *Mycobacterium avium* subspecies *paratuberculosis*. BMC Genomics 17:79. 10.1186/s12864-015-2234-5 26813574 PMC4729121

[B10] Cai G. Pauli G. F. Wang Y. Jaki B. U. Franzblau S. G. (2013). Rapid determination of growth inhibition of *Mycobacterium tuberculosis* by GC-MS/MS quantitation of tuberculostearic acid. Tuberculosis 93, 322–329. 10.1016/j.tube.2012.12.004 23454100

[B11] Chiodini R. J. Van Kruiningen H. J. (1985). Characterization of *Mycobacterium paratuberculosis* of bovine, caprine, and ovine origin by gas-liquid chromatographic analysis of fatty acids in whole-cell extracts. Am. J. Vet. Res. 46, 1980–1989. 4051303

[B12] Colbourn E. A. Rowe R. C. (2009). Novel approaches to neural and evolutionary computing in pharmaceutical formulation: challenges and new possibilities. Future Med. Chem. 1, 713–726. 10.4155/fmc.09.57 21426034

[B13] Collins D. M. Gabric D. M. de Lisle G. W. (1990). Identification of two groups of *Mycobacterium paratuberculosis* strains by restriction endonuclease analysis and DNA hybridization. J. Clin. Microbiol. 28, 1591–1596. 2166089 10.1128/jcm.28.7.1591-1596.1990 PMC267994

[B14] Dang N. A. Mourão M. Kuijper S. Walters E. Janssen H. G. Kolk A. H. (2015). Direct detection of *Mycobacterium tuberculosis* in sputum using combined solid phase extraction-gas chromatography-mass spectrometry. J. Chromatogr. B Analyt. Technol. Biomed. Life Sci. 986–987, 115–122. 10.1016/j.jchromb.2015.01.045 25728368

[B15] Everman J. L. Eckstein T. M. Roussey J. Coussens P. Bannantine J. P. Bermudez L. E. (2015). Characterization of the inflammatory phenotype of *Mycobacterium avium* subspecies *paratuberculosis* using a novel cell culture passage model. Microbiology 161, 1420–1434. 10.1099/mic.0.000106 25957310

[B16] Feller M. Huwiler K. Stephan R. Altpeter E. Shang A. Furrer H.. (2007). *Mycobacterium avium* subspecies *paratuberculosis* and Crohn's disease: a systematic review and meta-analysis. Lancet Infect. Dis. 7, 607–613. 10.1016/S1473-3099(07)70211-6 17714674

[B17] Fratti R. A. Chua J. Vergne I. Deretic V. (2003). *Mycobacterium tuberculosis* glycosylated phosphatidylinositol causes phagosome maturation arrest. Proc. Natl. Acad. Sci. U.S.A. 100, 5437–5442. 10.1073/pnas.0737613100 12702770 PMC154363

[B18] Gago J. Martínez-Núñez L. Landín M. Gallego P. P. (2010). Artificial neural networks as an alternative to the traditional statistical methodology in plant research. J. Plant. Physiol. 167, 23–27. 10.1016/j.jplph.2009.07.007 19716625

[B19] Gilleron M. Lindner B. Puzo G. (2006). MS/MS approach for characterization of the fatty acid distribution on mycobacterial phosphatidyl-myo-inositol mannosides. Anal. Chem. 78, 8543–8548. 10.1021/ac061574a 17165851

[B20] Gilleron M. Quesniaux V. F. J. Puzo G. (2003). Acylation state of the phosphatidylinositol hexamannosides from *Mycobacterium bovis* bacillus Calmette Guérin and *Mycobacterium tuberculosis* H37Rv and its implication in Toll-like receptor response. J. Biol. Chem. 278, 29880–29889. 10.1074/jbc.M303446200 12775723

[B21] Gilleron M. Ronet C. Mempe M. Monsarrat B. Gachelin G. Puzo G. (2001). Acylation state of the phosphatidylinositol mannosides from *Mycobacterium bovis* bacillus Calmette Guérin and ability to induce granuloma and recruit natural killer T cells. J. Biol. Chem. 276, 34896–34904. 10.1074/jbc.M103908200 11441009

[B22] Guerin M. E. Korduláková J. Alzari P. M. Brennan P. J. Jackson M. (2010). Molecular basis of phosphatidyl-myo-inositol mannoside biosynthesis and regulation in mycobacteria. J. Biol. Chem. 285, 33577–33583. 10.1074/jbc.R110.168328 20801880 PMC2962455

[B23] Juste R. A. Elguezabal N. Garrido J. M. Pavon A. Geijo M. V. Sevilla I.. (2008). On the prevalence of *M. avium* subspecies *paratuberculosis* DNA in the blood of healthy individuals and patients with inflammatory bowel disease. PLoS ONE 3:e2537. 10.1371/journal.pone.0002537 18596984 PMC2434204

[B24] Juste R. A. Elguezabal N. Pavón A. Garrido J. M. Geijo M. Sevilla I.. (2009). Association between *Mycobacterium avium* subsp. *paratuberculosis* DNA in blood and cellular and humoral immune response in inflammatory bowel disease patients and controls. Int. J. Infect. Dis. 13, 247–254. 10.1016/j.ijid.2008.06.034 18922720

[B25] Kolattukudy P. E. Fernandes N. D. Azad A. K. Fitzmaurice A. M. Sirakova T. D. (1997). Biochemistry and molecular genetics of cell-wall lipid biosynthesis in mycobacteria. Mol. Microbiol. 24, 263–270. 9159514 10.1046/j.1365-2958.1997.3361705.x

[B26] Lambert M. A. Moss C. W. Silcox V. A. Good R. C. (1986). Analysis of mycolic acid cleavage products and cellular fatty acids of *Mycobacterium* species by capillary gas chromatography. J. Clin. Microbiol. 23, 731–736. 3084554 10.1128/jcm.23.4.731-736.1986 PMC362826

[B27] Landín M. Rowe R. C. York P. (2009). Advantages of neurofuzzy logic against conventional experimental design and statistical analysis in studying and developing direct compression formulations. Eur. J. Pharm. Sci. 38, 325–331. 10.1016/j.ejps.2009.08.004 19716414

[B28] Li Y. Powell D. A. Shaffer S. A. Rasko D. A. Pelletier M. R. Leszyk J. D.. (2012). LPS remodeling is an evolved survival strategy for bacteria. Proc. Natl. Acad. Sci. U.S.A. 109, 8716–8721. 10.1073/pnas.1202908109 22586119 PMC3365160

[B29] Luquin M. Ausina V. López Calahorra F. Belda F. García Barceló M. Celma C.. (1991). Evaluation of practical chromatographic procedures for identification of clinical isolates of mycobacteria. J. Clin. Microbiol. 29, 120–130. 1993746 10.1128/jcm.29.1.120-130.1991 PMC269715

[B30] Majumder N. Bhattacharjee S. Dey R. Bhattacharyya Majumdar S. Pal N. K. Majumdar S. (2008). Arabinosylated lipoarabinomannan modulates the impaired cell mediated immune response in *Mycobacterium tuberculosis* H37Rv infected C57BL/6 mice. Microbes Infect. 10, 349–357. 10.1016/j.micinf.2007.12.013 18417403

[B31] Mukhopadhyay S. Nair S. Ghosh S. (2012). Pathogenesis in tuberculosis: transcriptomic approaches to unraveling virulence mechanisms and finding new drug targets. FEMS Microbiol. Rev. 36, 463–485. 10.1111/j.1574-6976.2011.00302.x 22092372

[B32] Naser S. A. Sagramsingh S. R. Naser A. S. Thanigachalam S. (2014). *Mycobacterium avium* subspecies *paratuberculosis* causes Crohn's disease in some inflammatory bowel disease patients. World J. Gastroenterol. 20, 7403–7415. 10.3748/wjg.v20.i23.7403 24966610 PMC4064085

[B33] Odham G. Larsson L. Mårdh P. A. (1979). Demonstration of tuberculostearic acid in sputum from patients with pulmonary tuberculosis by selected ion monitoring. J. Clin. Invest. 63, 813–819. 10.1172/JCI109380 109465 PMC372021

[B34] Olivier M. Berthon P. Chastang J. Cordier G. Lantier F. (2001). Establishment and characterisation of ovine blood monocyte-derived cell lines. Vet. Immunol. Immunopathol. 82, 139–151. 10.1016/S0165-2427(01)00330-0 11587730

[B35] Ott S. L. Wells S. J. Wagner B. A. (1999). Herd-level economic losses associated with Johne's disease on US dairy operations. Prev. Vet. Med. 40, 179–192. 10423773 10.1016/s0167-5877(99)00037-9

[B36] Ozbek A. Aktas O. (2003). Identification of three strains of *Mycobacterium* species isolated from clinical samples using fatty acid methyl ester profiling. J. Int. Med. Res. 31, 133–140. 10.1177/147323000303100210 12760317

[B37] Rengarajan J. Bloom B. R. Rubin E. J. (2005). Genome-wide requirements for Mycobacterium tuberculosis adaptation and survival in macrophages. Proc Natl Acad Sci U.S.A. 102, 8327–8332. 10.1073/pnas.0503272102 15928073 PMC1142121

[B38] Rivière M. Moisand A. Lopez A. Puzo G. (2004). Highly ordered supra-molecular organization of the mycobacterial lipoarabinomannans in solution. Evidence of a relationship between supra-molecular organization and biological activity. J. Mol. Biol. 344, 907–918. 10.1016/j.jmb.2004.09.092 15544801

[B39] Sasser M. (1990). Identification of bacteria by gas chromatography of cellular fatty acids. MIDI Technical Note.

[B40] Scanu A. M. Bull T. J. Cannas S. Sanderson J. D. Sechi L. A. Dettori G.. (2007). *Mycobacterium avium* subspecies *paratuberculosis* infection in cases of irritable bowel syndrome and comparison with Crohn's disease and Johne's disease: common neural and immune pathogenicities. J. Clin. Microbiol. 45, 3883–3890. 10.1128/JCM.01371-07 17913930 PMC2168579

[B41] Sevilla I. A. Singh S. V. Garrido J. M. Aduriz G. Rodríguez S. Geijo M. V.. (2005). Molecular typing of *Mycobacterium avium* subspecies *paratuberculosis* strains from different hosts and regions. Rev. Sci. Tech. 24, 1061–1066. 10.20506/rst.24.3.1634 16642774

[B42] Sevilla I. Garrido J. M. Geijo M. Juste R. A. (2007). Pulsed-field gel electrophoresis profile homogeneity of *Mycobacterium avium* subsp. *paratuberculosis* isolates from cattle and heterogeneity of those from sheep and goats. BMC Microbiol. 7:18. 10.1186/1471-2180-7-18 17352818 PMC1832200

[B43] Shao Q. Rowe R. C. York P. (2006). Comparison of neurofuzzy logic and neural networks in modelling experimental data of an immediate release tablet formulation. Eur. J. Pharm. Sci. 28, 394–404. 10.1016/j.ejps.2006.04.007 16781126

[B44] Souza C. D. Evanson O. A. Sreevatsan S. Weiss D. J. (2007). Cell membrane receptors of bovine mononuclear phagocytes involved on phagocytosis of *Mycobcaterium avium* subsp. paratuberculosis. Am. J. Vet. Res. 68, 975–980. 10.2460/ajvr.68.9.975 17764412

[B45] Souza C. Davis W. C. Eckstein T. M. Sreevatsan S. Weiss D. J. (2013). Mannosylated lipoarabinomannans from *Mycobacterium avium* subsp. *paratuberculosis* alters the inflammatory response by bovine macrophages and suppresses killing of *Mycobacterium avium* subsp. *avium* organisms. PLoS ONE 8:e75924. 10.1371/journal.pone.0075924 24098744 PMC3786972

[B46] Stabel J. R. Stabel T. J. (1995). Immortalization and characterization of bovine peritoneal macrophages transfected with SV40 plasmid DNA. Vet. Immunol. Immunopathol. 45, 211–220. 7676607 10.1016/0165-2427(94)05348-v

[B47] Thirunavukkarasu S. de Silva K. Plain K. M. Whittington R. (2014). Role of host- and pathogen-associated lipids in directing the immune response in mycobacterial infections, with emphasis on *Mycobacterium avium* subsp. paratuberculosis. Crit. Rev. Microbiol. 42, 262–275. 10.3109/1040841X.2014.932327 25163812

[B48] Torrelles J. B. Azad A. K. Schlesinger L. S. (2006). Fine discrimination in the recognition of individual species of phosphatidyl-myo-inositol mannosides from *Mycobacterium tuberculosis* by C-type lectin pattern recognition receptors. J. Immunol. 177, 1805–1816. 10.4049/jimmunol.177.3.1805 16849491

[B49] Torrelles J. B. Knaup R. Kolareth A. Slepushkina T. Kaufman T. M. Kang P.. (2008). Identification of *Mycobacterium tuberculosis* clinical isolates with altered phagocytosis by human macrophages due to a truncated lipoarabinomannan. J. Biol. Chem. 283, 31417–31428. 10.1074/jbc.M806350200 18784076 PMC2581576

[B50] Torrelles J. B. Schlesinger L. S. (2010). Diversity in *Mycobacterium tuberculosis* mannosylated cell wall determinants impacts adaptation to the host. Tuberculosis 90, 84–93. 10.1016/j.tube.2010.02.003 20199890 PMC2855779

