# Supplementary figures and images for: Mycobacterium avium subsp. paratuberculosis (Map) Fatty Acids Profile Is Strain-Dependent and Changes Upon Host Macrophages Infection
Source: Front Cell Infect Microbiol. 2017 Mar 21;7:89. doi: 10.3389/fcimb.2017.00089 (PMC5359295; doi:10.3389/fcimb.2017.00089)

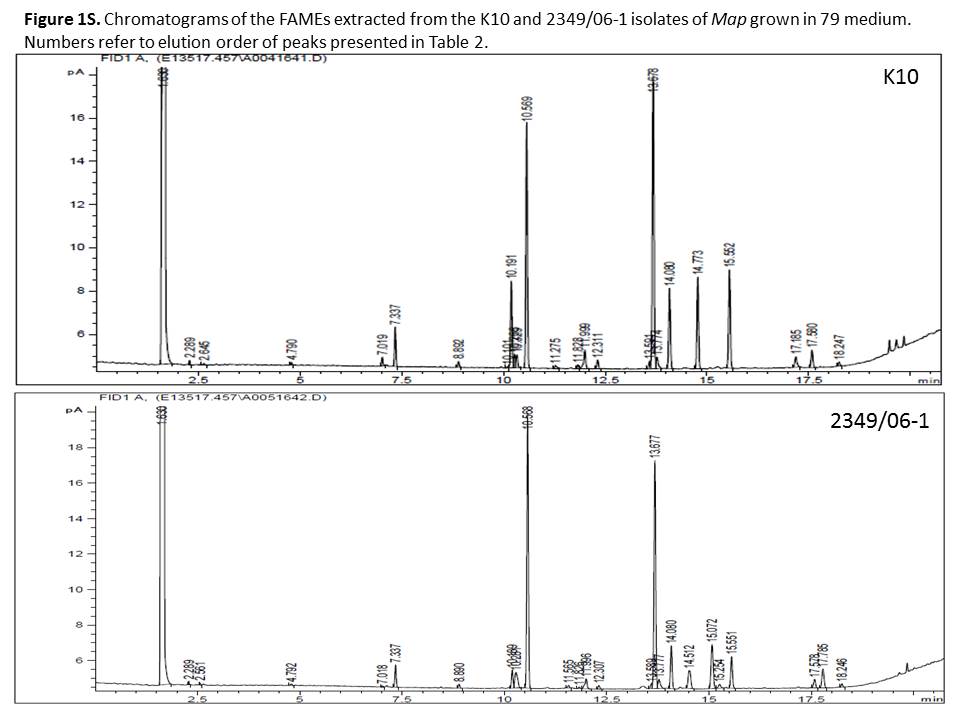

Supplement: Supplementary file 1 [file Image1.jpg]

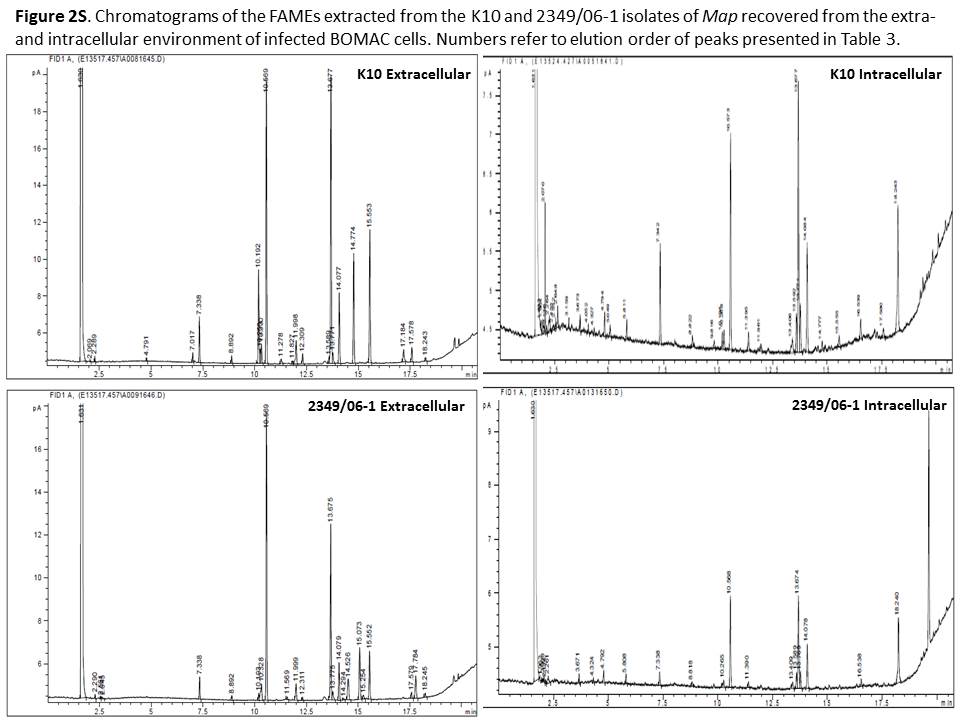

Supplement: Supplementary file 2 [file Image2.jpg]

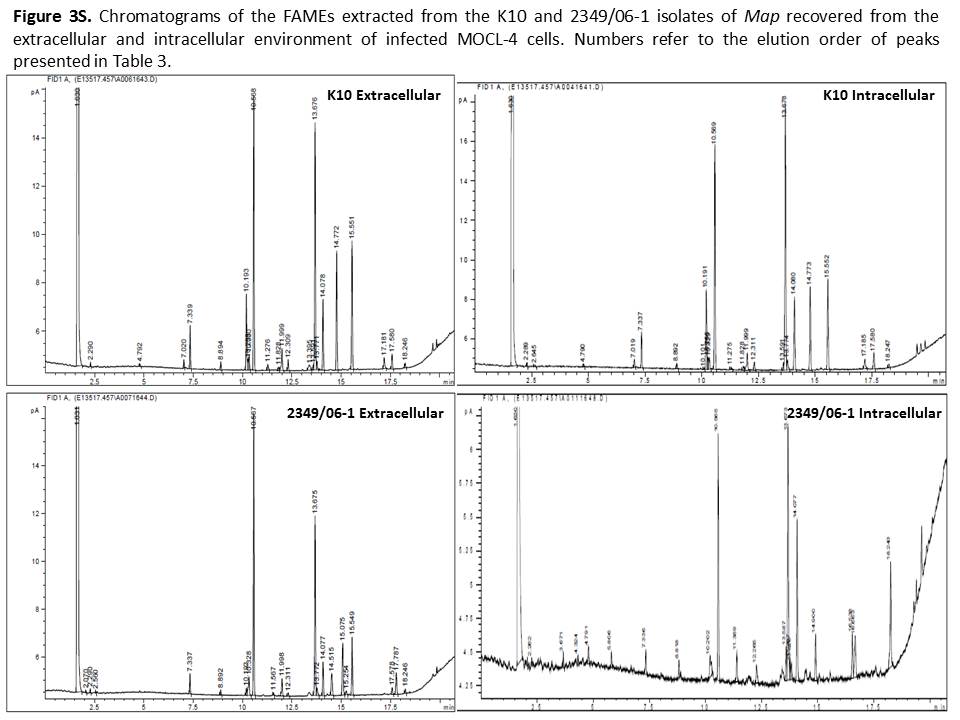

Supplement: Supplementary file 3 [file Image3.jpg]
